# Volumetric photoacoustic imaging of elastin and age-related remodeling using near-infrared probe ElaNIR^☆^

**DOI:** 10.1016/j.pacs.2026.100857

**Published:** 2026-07-18

**Authors:** Hyunseo Jeon, Jiwoong Kim, Haw-Young Kwon, Jihye Lee, Won Jong Kim, Nam-Young Kang, Young-Tae Chang, Chulhong Kim

**Affiliations:** a Department of Electrical Engineering, Convergence IT Engineering, Medical Science and Engineering, Institute of Artificial Intelligence, and Medical Device Innovation Center, Pohang University of Science and Technology, Republic of Korea; b Department of Chemistry, Pohang University of Science and Technology (POSTECH), Pohang 37673, Republic of Korea; c Department of Convergence IT Engineering, Pohang University of Science and Technology (POSTECH), Pohang 37673, Republic of Korea; d SenPro, Pohang University of Science and Technology, C5 building, Pohang, Gyeongbuk 37673, Korea

**Keywords:** Photoacoustic imaging, Photoacoustic computed tomography, Multispectral imaging, Elastin, Aging

## Abstract

Elastin supports tissue elasticity and structural stability, but its regeneration is minimal in adulthood. Monitoring elastin remodeling is therefore important for understanding aging and related cardiovascular disease. However, fluorescence imaging lacks penetration for deep-tissue visualization, while histological methods are invasive, endpoint-based, and limited in volumetric assessment. Here, we demonstrate 3D multispectral photoacoustic computed tomography (PACT) for volumetric assessment of ElaNIR, a near-infrared elastin-targeted probe. Multispectral unmixing separates ElaNIR signals from endogenous hemoglobin, enabling depth-resolved visualization of probe distribution within anatomical context. Young mice showed strong, uniform ElaNIR signals in elastin-rich tissues, including skin and ear, whereas aged mice showed reduced and discontinuous signals. In contrast, aged mice exhibit increased ElaNIR signals in clearance-related organs, including kidneys, liver, and bladder, suggesting altered probe retention and biodistribution with aging. These results establish 3D multispectral PACT as a volumetric imaging framework that integrates elastin-associated probe retention with organ-level biodistribution for interpreting age-related changes.

## 1 Introduction

Elastin is an essential extracellular matrix (ECM) protein providing elasticity and structural stability to tissues, including skin, arteries, and elastic organs [1], [2]. Because elastin synthesis declines substantially after early development and its turnover is extremely slow [3], [4], [5], age-related fragmentation, thinning, and disorganization of elastic fibers accumulate over time and serve as indicators of cumulative tissue remodeling, cardiovascular dysfunction, and impaired tissue regeneration [6], [7], [8], [9], [10], [11]. Consequently, imaging approaches that can noninvasively visualize elastin biodistribution in vivo are important for understanding age‑related changes and linking microscopic matrix alterations to macroscopic tissue appearance and mechanics.

Conventional elastin imaging strategies have largely relied on histological staining and surface‑weighted optical techniques. Verhoeff–Van Gieson and related elastin stains provide high-resolution visualization of elastic fibers, but require tissue excision, fixation, and sectioning, thereby precluding longitudinal in vivo monitoring [12], [13], [14]. Label-free optical approaches based on elastin autofluorescence can resolve elastic lamellae and fiber architecture [15], [16], [17]. However, signal overlap with other endogenous fluorophores, tissue scattering, and limited penetration depth can reduce signal-to-background contrast, making it challenging to distinguish elastin in complex intravital environments. To improve molecular contrast, several elastin-targeted fluorescent probes, such as sulforhodamine B, Col-F, and Alexa Fluor 633, have been developed [18], [19], [20]. Nevertheless, their in vivo utility remains limited by their short emission wavelengths, as tissue scattering and optical attenuation restrict imaging depth to a few hundred micrometers. These considerations motivate an in vivo imaging framework that can capture depth-resolved probe signals and support the interpretation of elastin-associated signal distribution beyond the superficial limits of conventional fluorescence (FL) imaging.

These requirements point to photoacoustic (PA) imaging, which converts optical absorption into ultrasound signals to provide high spatial resolution in deep tissues [21], [22], [23], [24], [25], [26]. Its sensitivity to optical absorption enables in vivo visualization of molecular probe signals while simultaneously mapping endogenous chromophores such as hemoglobin [27], [28], [29], [30], [31], [32], [33], [34], [35], [36], [37]. Furthermore, multispectral PA imaging combined with spectral unmixing can improve the separation of exogenous probe signals from dominant background absorbers [38], [39], [40], [41], [42], [43]. This approach supports depth-resolved molecular analysis by reducing background interference and providing anatomical context. Within this framework, ElaNIR, a near-infrared (NIR) elastin-affinity probe, is particularly suitable for PA-based elastin imaging because its strong NIR absorption enables efficient deep-tissue PA signal acquisition. ElaNIR retains the key physicochemical features of the ZW800–1 scaffold, including low serum protein binding, minimal nonspecific background, and rapid renal clearance. The elastin-targeting capability of ElaNIR was previously supported by histological cross-validation [44]. In cryosections of elastin-rich tissues, including the lungs, skin, and urinary bladder, ElaNIR FL signals showed spatial patterns consistent with the immunohistochemical (IHC) staining patterns of an anti-elastin antibody. These results confirm that ElaNIR preferentially labels elastin-containing fiber structures in tissue. In contrast, ElaNIR signals did not overlap with anti-collagen antibody staining, indicating that the observed retention is driven by elastin-specific binding rather than non-specific accumulation within the ECM. Structurally, ElaNIR incorporates an additional biphenyl moiety that may enhance hydrophobic interactions with elastin-rich fiber structures. A previous study demonstrated the feasibility of ElaNIR-based PA imaging by detecting age-dependent differences in skin PA signals between young and old mice [44]. However, this prior demonstration was primarily limited to localized skin analysis based on single-wavelength PA intensity, without organ-level volumetric assessment or multispectral separation of ElaNIR signals.

In this study, we address these limitations by employing 3D multispectral photoacoustic computed tomography (PACT) for the volumetric assessment of ElaNIR signals in vivo. We utilize a PACT system with a 1024-element hemispherical transducer array for efficient omnidirectional signal detection and isotropic 3D reconstruction [45], [46]. The system supports whole-body small-animal imaging to depths of approximately 10 mm and has been validated in multiple preclinical disease models [46], [47], [48], [49]. We first characterized the spectral properties and detection sensitivity of ElaNIR to validate its suitability as a PA contrast agent. We then administered the probe to mice and acquired multispectral images in the NIR region selected based on the absorption profile of ElaNIR. This strategy enables spectral unmixing of ElaNIR signals from endogenous hemoglobin, allowing organ-level 3D visualization and quantitative analysis of ElaNIR signal distribution within an anatomical context. Using this platform, we compared young and aged mice to evaluate age-dependent changes in the 3D distribution of ElaNIR across elastin-rich tissues and major clearance-related organs. Collectively, this study establishes 3D multispectral PACT as a volumetric imaging framework for assessing ElaNIR signals in vivo to support the interpretation of elastin-associated signal distribution and age-related changes.

## 2 Materials and methods

### 2.1 ElaNIR synthesis

ElaNIR (CyZW−599), an elastin-targeted near-infrared zwitterionic probe based on the ZW800–1 scaffold, was synthesized according to a previously established CyZW library protocol [44]. Briefly, a set of primary amines was reacted with tert-butyl 2-bromoacetate in acetonitrile in the presence of DIEA to generate the corresponding amine-derived intermediates. The resulting intermediates were then coupled to the ZW800–1 scaffold using HATU and DIEA in a DMSO/DMF (1:4) solvent mixture to form CyZW precursors. Following purification, the tert-butyl protecting group was removed under acidic conditions (20% TFA in dichloromethane) to yield the zwitterionic CyZW analogs. ElaNIR (CyZW−599) was obtained after final purification and confirmed by NMR and spectroscopic analysis as described previously [44].

### 2.2 3D multispectral photoacoustic computed tomography

Fig. S1 shows the configuration of the multispectral photoacoustic computed tomography system used in this study. Optical excitation was provided by a tunable optical parametric oscillator (OPO) laser (PhotoSonus M−20, Ekspla, Lithuania) operating over a wavelength range of 690–1064 nm. The laser beam was delivered to the target through a customized fiber bundle (Opotek, USA). The generated photoacoustic signals were detected using a 1024-channel hemispherical ultrasound transducer array (Japan Probe, Inc., Japan; 2 MHz center frequency; 54% bandwidth) and acquired with a data acquisition (DAQ) system (Vantage 256, Verasonics, USA). 3D volumetric images were reconstructed using a customized phase-rotation beamforming algorithm implemented in CUDA and MATLAB. Whole-body imaging of small animals was accomplished by performing raster scanning with a 3-axis motorized stage (LSQ150A, Zaber, USA), followed by image stitching of each single volume image. To enable precise analysis of the acquired 3D volumetric data, cross-sectional analysis and skin extraction were performed using 3D PHOVIS software [50]. For cross-sectional visualization, we utilized a local maximum amplitude projection (MAP) technique that projects signal over a narrow, user-defined depth range. Unlike conventional MAP, which projects the entire volume, this approach enables cross-sectional visualization by enhancing signals from the skin layer. For skin extraction, a 3D skin surface profile was first generated based on the strong PA signals at the skin surface in 3D PHOVIS. We then reconstructed a skin MAP image by selectively projecting signals from the depth range corresponding to the skin layer relative to the generated surface profile.

### 2.3 Measurement of ElaNIR properties

The absorption spectrum of ElaNIR was measured using a microplate-based spectrophotometer (SpectraMax M5, Molecular Devices). ElaNIR was dissolved in PBS, followed by serial dilution to achieve final concentrations of 31.25, 62.5, 125, 250, and 500 µM. Each sample was dispensed into a 96-well flat, clear-bottom microplate (Greiner). Absorption spectra were recorded at room temperature over a wavelength range of 600–970 nm, using PBS-only wells as blanks for background correction.

To measure the PA spectrum of ElaNIR, solutions were prepared by dissolving ElaNIR in PBS at concentrations of 31.3, 62.5, 125, 250, and 500 µM. Each solution was loaded into a 1.5 mL centrifuge tube. Multispectral PACT imaging was then performed across a wavelength range of 670–910 nm with a step size of 20 nm. To minimize bias arising from the region of interest (ROI) selection, the mean PA signal was calculated from three independently defined ROIs within each concentration phantom and used as the representative value.

To measure the in vivo detection sensitivity of ElaNIR, a subcutaneous injection-based sensitivity test was performed using Balb/c nude mice. ElaNIR solutions were prepared at serial concentrations ranging from 3.91 to 500 µM (3.91, 7.81, 15.6, 31.3, 62.5, 125, 250, 500 µM). A volume of 50 µL of each concentration was subcutaneously injected into the right flank of the mice. PACT images of the right sagittal plane were acquired at 800 nm under identical experimental conditions. The mean PA signal intensity was calculated from three independently defined 3D ROIs at the injection site. The background signal was quantified from signal-free regions, and the detection threshold was defined as the mean background signal plus three times its standard deviation. A calibration curve relating probe concentration to PA signal amplitude was obtained using log–linear regression. The LOD was defined as the concentration corresponding to the intersection between the fitted calibration curve and the detection. Finally, single-pixel sensitivity was derived by calculating the volume of a single pixel based on the system’s isotropic resolution of 380 µm and multiplying this volume by the threshold concentration to estimate the minimum detectable amount of ElaNIR per pixel.

### 2.4 In vivo experimental procedure

For general in vivo analysis, 6-week-old Balb/c nude mice were used. For age-dependent comparative imaging, 6-week-old and 12-month-old Balb/c nude mice were used as young and aged groups, respectively. During imaging, a customized mask connected to an isoflurane anesthesia system (VIP3000 Veterinary Vaporizer, Midmark, USA) was used to maintain anesthesia. The mice were positioned on a 3D-printed mouse holder and submerged in a water tank to ensure acoustic coupling. A water circulator (C-WBL, Changshin Science, Republic of Korea) was employed to maintain a stable body temperature throughout the imaging procedure.

ElaNIR was systemically administered by retro-orbital injection. Briefly, mice were anesthetized with isoflurane and placed in a supine position. A 1 mM ElaNIR solution prepared in PBS was injected into the retro-orbital venous sinus at a dose of 10 μL/g. For control imaging, CyZW−274 was prepared in PBS at the same concentration and administered using the same injection route and dose. The needle was gently advanced into the retro-orbital venous sinus at the medial canthus, and the solution was slowly injected to minimize reflux and tissue damage. After injection, gentle pressure was applied to the eyelid for several seconds to achieve hemostasis [51].

To validate the observations from PACT imaging, in vivo fluorescence imaging was performed after PACT monitoring. Specifically, whole-body fluorescence images of the corresponding young and aged mice were acquired immediately after 1 h post-injection PACT monitoring using an IVIS imaging system (PerkinElmer, USA). Image acquisition and analysis were performed using Living Image software (PerkinElmer, USA).

All animal studies were carried out following protocols approved by the Institutional Animal Care and Use Committee (IACUC) of Pohang University of Science and Technology (POSTECH). After completion of the in vivo imaging experiments, the mice were sacrificed in accordance with the institutional guidelines approved by POSTECH.

### 2.5 Spectral unmixing and quantitative analysis

To separate the ElaNIR signals from the multispectral PACT data, spectral unmixing was applied. Multispectral PACT images were acquired at three excitation wavelengths (700, 800, and 900 nm), and the signal intensity at each wavelength was compensated using the corresponding measured laser pulse energy (ES220C, Thorlabs, USA). These wavelengths were selected based on the spectral contrast between ElaNIR and hemoglobin. ElaNIR shows strong absorption and PA signal generation at 700–800 nm but negligible absorption around 900 nm. The 800-nm wavelength is also close to the isosbestic point of oxy- and deoxy-hemoglobin. Thus, the selected wavelength set provides ElaNIR-sensitive wavelengths and an ElaNIR-off wavelength for separating ElaNIR signals from endogenous hemoglobin signals. Because spectral unmixing requires the absorption spectrum of each component, the in vivo spectrum of ElaNIR was experimentally estimated. In the pre-injection PACT images, the bladder region shows negligible signals comparable to the background level, indicating minimal endogenous PA contribution. After ElaNIR injection, strong signals are observed in the bladder due to renal clearance and accumulation of ElaNIR. Therefore, the bladder provides a suitable anatomical region for estimating the in vivo reference spectrum of ElaNIR with relatively low contribution from endogenous absorbers and strong ElaNIR-dominant contrast. Based on this rationale, a 3D ROI was defined for the bladder in the multispectral dataset **(**Fig. S2a, Supporting information**)**, and the energy-compensated PA signal within the ROI was quantified to estimate the in vivo spectrum of ElaNIR **(**Fig. S2b, Supporting information**)**. Least-squares fitting-based spectral unmixing was then performed using the estimated ElaNIR spectrum together with the known spectra of oxygenated hemoglobin and deoxygenated hemoglobin to reconstruct three-dimensional distribution maps of oxygenated hemoglobin (HbO), deoxygenated hemoglobin (HbR), and ElaNIR:

(1) $$\left[\begin{array}{ccc} \varepsilon_{H\mathrm{bO}}^{700} & \varepsilon_{H\mathrm{bR}}^{700} & \varepsilon_{E\mathrm{laNIR}}^{700}\\ \varepsilon_{H\mathrm{bO}}^{800} & \varepsilon_{H\mathrm{bR}}^{800} & \varepsilon_{E\mathrm{laNIR}}^{800}\\ \varepsilon_{H\mathrm{bO}}^{900} & \varepsilon_{H\mathrm{bR}}^{900} & \varepsilon_{E\mathrm{laNIR}}^{900} \end{array}\right]\left[\begin{array}{c} C_{\mathrm{HbO}}\\ \begin{array}{c} C_{\mathrm{HbR}}\\ C_{\mathrm{ElaNIR}} \end{array} \end{array}\right]=\left[\begin{array}{c} {\mathrm{PA}}_{700}\\ {\mathrm{PA}}_{800}\\ {\mathrm{PA}}_{900} \end{array}\right]$$.

In the above equation, C is the concentration of each component, PA is the fluence-compensated PA signal amplitude, and ε is the molar extinction coefficient of each component at each wavelength. The total hemoglobin (HbT) volume was obtained by voxel-wise summation of the unmixed HbO and HbR components. For visualization, the unmixed ElaNIR signal (green colormap) and HbT signal (hot colormap) were superimposed on the 800 nm PACT image displayed in grayscale. To validate the spectral unmixing procedure using the estimated ElaNIR spectrum, ElaNIR was subcutaneously injected into the right flank of a nude mouse. Multispectral PACT images were acquired and unmixed using the same spectral unmixing procedure (Fig. S2c**-d**, Supporting information).

For quantitative analysis of ElaNIR PA signals, volumes of interest were manually defined. To accurately extract the 3D volumes, we first defined a rectangular ROI on the XY-MAP images and extended it along the z-axis to obtain the corresponding 3D sub-volume. We then delineated polygonal ROIs along the skin and organ boundaries on the XZ-MAP images to isolate the 3D volumes for the skin and each major organ. To optimally quantify signals derived from the exogenous probe, the statistical analysis was restricted to the top 10–30% of pixels with the highest PA intensities within each ROI. This range was selected based on previous studies to minimize the effects of overshooting and noise. All PACT results were obtained from experiments performed in three mice, and all data are presented as mean values ± standard error of the mean. A priori power analysis was performed using G*Power to determine the required sample size for comparing ElaNIR-associated PA signals between young and aged mice [52], [53]. Statistical significance was assessed using a two-tailed *t*-test. Significance levels are indicated as *p < 0.05, **p < 0.01, or ***p < 0.005.

## 3 Results

### 3.1 Characterization of ElaNIR: elastin-specific NIR probe for PACT

**Fig. 1:**
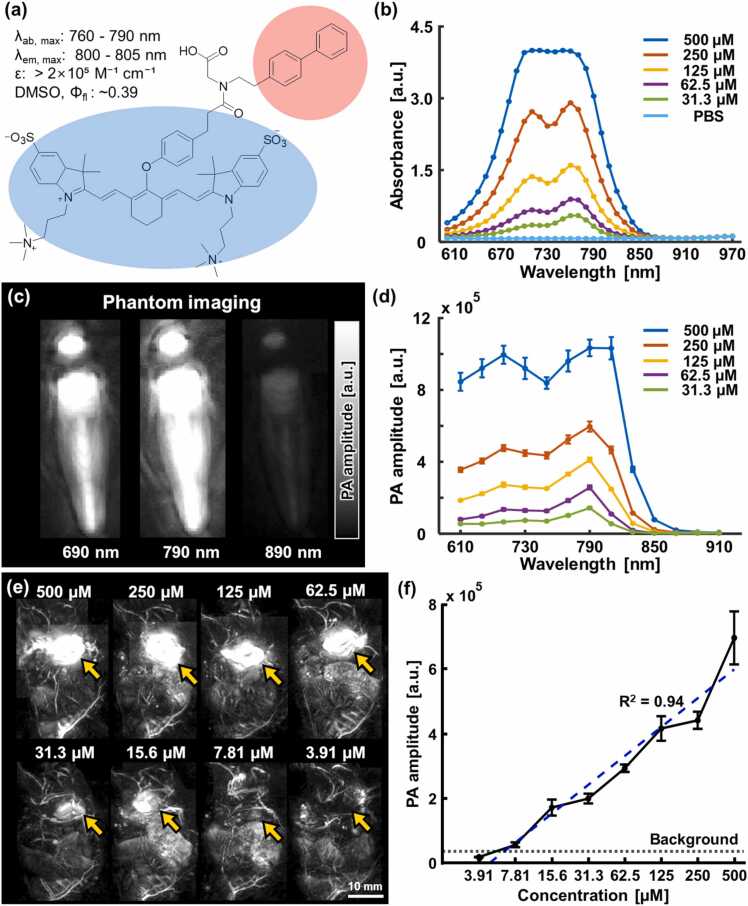
**Molecular design and properties of ElaNIR.** (a) Structural domains of ElaNIR (CyZW−599) and their functional relevance. ElaNIR contains two functionally distinct regions: a hydrophobic biphenyl aromatic head (red), which contributes to selective association with elastin-rich hydrophobic domains, and a zwitterionic NIR fluorophore core (blue), which provides near-infrared optical properties and low nonspecific binding. Optical parameters and structural origin of ElaNIR are adapted from Su et al. [44]. (b) Absorption spectra of ElaNIR measured at different concentrations. (c) ElaNIR phantom images of a 62.5 µM solution acquired at 690, 790, and 890 nm. (d) PA spectra of ElaNIR measured at different concentrations (n = 3 ROIs per concentration). (e) Right sagittal plane MAP images acquired after subcutaneous injection of ElaNIR at different concentrations. Orange arrows indicate the injection site. (f) Concentration-dependent PA signal amplitude of ElaNIR (n = 3 ROIs per concentration). Quantified PA signals were fitted with a regression analysis (R² = 0.94). The intersection with the background level (dotted gray line) was used to estimate the limit of detection. Error bars represent standard errors.

We first evaluated the optical and photoacoustic properties of ElaNIR to determine its suitability as a molecular contrast agent for PACT. Structurally, ElaNIR retains the CyZW core scaffold and incorporates an additional biphenyl moiety designed to enhance elastin affinity **(**Fig. 1**a)**. ElaNIR exhibits strong NIR absorption with a peak around 760–790 nm and fluorescence emission around 800–805 nm, supporting its use for wavelength-dependent PA contrast generation in the NIR window **(**Fig. 1**b)**. Its high molar extinction coefficient (> 2 × 10⁵ M⁻¹ cm⁻¹) provides strong optical absorption for PA signal generation, and its quantum yield of approximately 0.39 supports the utility of ElaNIR for multimodal fluorescence and PA imaging. Additionally, the zwitterionic charge distribution of the CyZW scaffold is expected to reduce nonspecific tissue accumulation and background signals in vivo, facilitating spectral unmixing by improving the separation of ElaNIR from endogenous absorbers [54], [55].

To characterize the PA properties of ElaNIR, we performed phantom imaging using ElaNIR solutions at varying concentrations (31.3–500 µM). Phantom images acquired from a 62.5 µM ElaNIR solution show that PA signals at 690, 790, and 890 nm are clearly distinguishable **(**Fig. 1**c)**. The measured PA spectra show a peak at 790 nm, consistent with the optical absorption profile of ElaNIR, and a linear increase in PA amplitude with concentration **(**Fig. 1**d)**. Subsequently, to assess in vivo detection sensitivity, we subcutaneously injected serially diluted ElaNIR solutions (3.91–500 µM**)** into the right flank of mice and imaged them at 800 nm **(**Fig. 1**e)**. The quantified PA amplitude increases with probe concentration over a wide dynamic range, exhibiting a strong correlation (R² = 0.94). The signal remains clearly distinguishable from the background even at low micromolar concentrations, yielding a limit of detection (LOD) of 6.33 µM **(**Fig. 1**f)**. This corresponds to a single-pixel sensitivity of 0.35 pmol based on the system’s 380 µm isotropic spatial resolution, demonstrating the high detection sensitivity of ElaNIR for quantitative molecular PACT imaging.

### 3.2 In vivo 3D multispectral PACT and spectral unmixing of ElaNIR signals

**Fig. 2:**
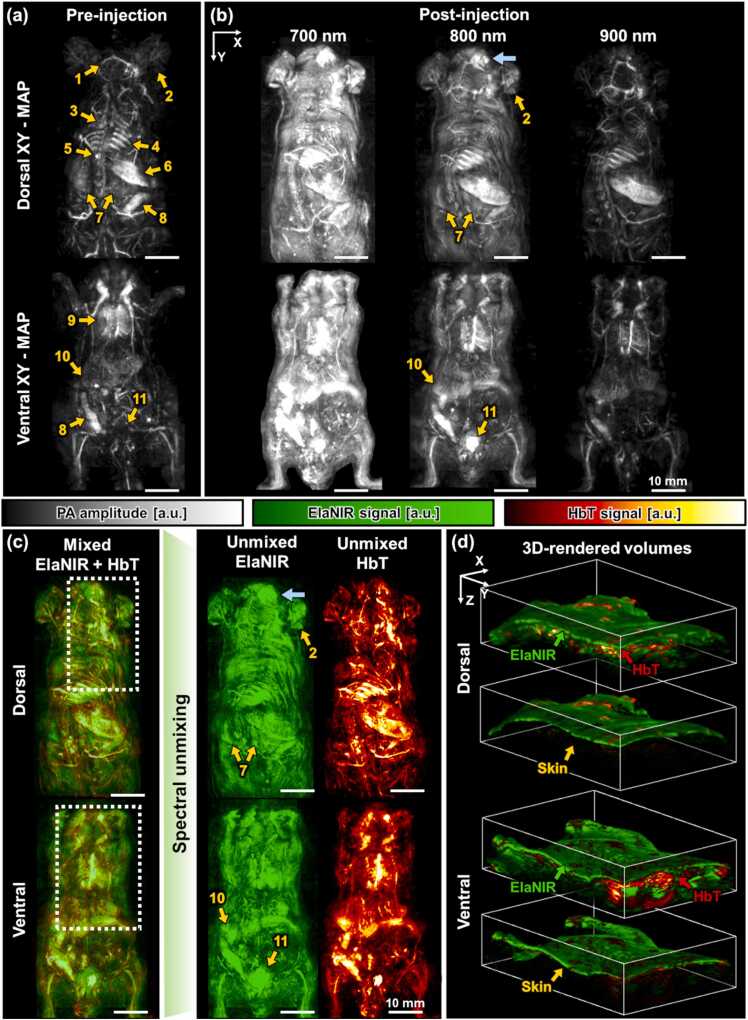
**3D multispectral PACT and spectral unmixing of ElaNIR signals.** (a) Pre-injection whole-body MAP images acquired in dorsal and ventral views at 800 nm. (b) Post-injection whole-body MAP images acquired in the dorsal and ventral planes at 700, 800, and 900 nm following ElaNIR administration. (c) Mixed MAP images and spectrally unmixed MAP images of ElaNIR (green) and HbT (hot) in the dorsal and ventral planes. Orange arrows indicate major organs. Sky blue arrows indicate residual signal near the administration site. (d) 3D volumetric analysis of unmixed ElaNIR and HbT signals. Volumes correspond to the white dashed-box region in panel (c). 1, transverse sinuses; 2, ear; 3, brown adipose tissue; 4, rib; 5, spine; 6, spleen; 7, kidney; 8, intestine; 9, heart; 10, liver; and 11, bladder.

To visualize the in vivo distribution of ElaNIR signals in mice, we performed whole-body PACT after retro-orbital injection of ElaNIR (1 mM, 10 µL/g). The PACT system utilizes a 1024-element hemispherical transducer array that efficiently captures PA waves propagating in all directions, enabling isotropic spatial resolution for 3D small-animal imaging (Fig. S1, Supporting information)**.** Multispectral images were acquired at 700, 800, and 900 nm to spectrally separate ElaNIR signals from endogenous hemoglobin signals (deoxy- and oxy-hemoglobin). Pre-injection whole-body MAP images acquired at 800 nm clearly delineate vascular structures and major organs, serving as anatomical references for subsequent analyses (Fig. 2**a**). The anatomical features indicated by orange arrows correspond to the transverse sinuses (1), ear (2), brown adipose tissue (3), rib (4), spine (5), spleen (6), kidneys (7), intestine (8), heart (9), liver (10), and bladder (11). Following ElaNIR administration, whole-body MAP images show wavelength-dependent signal patterns (Fig. 2**b**). At 900 nm, vascular and organ anatomy dominate the images, whereas at 700 and 800 nm, strong ElaNIR signals are superimposed on the anatomical background, consistent with probe accumulation in superficial tissue regions. To assess whether these superficial ElaNIR signals are associated with elastin affinity rather than nonspecific dye accumulation, we performed control imaging using CyZW−274, a structurally analogous probe with low elastin affinity (Fig. S3, Supporting information). Under identical experimental conditions, CyZW−274 does not show comparable signals in elastin-rich tissues, such as the skin and ear. Signals in the periocular region likely correspond to injection-site residues (sky blue arrows).

Fig. 2**c** presents mixed MAP images and the corresponding spectrally unmixed MAP images of ElaNIR (green) and HbT (hot). In the mixed images, probe-derived signals overlap with hemoglobin-derived background signals, complicating the differentiation of ElaNIR signals from vascular structures. Spectral unmixing resolves this overlap by separating ElaNIR signals from the HbT-based anatomical background. As a result, the ElaNIR map highlights signals in the skin layers and selected anatomical regions including the ear, kidneys, liver, and bladder, whereas the HbT map delineates vasculature and organ morphology in both dorsal and ventral views. The spatial overlap between the ElaNIR and HbT maps may indicate regions where ElaNIR signals arising from elastin-bound probe and systemically circulating probe coincide with HbT-defined vascularized structures.

Fig. 2**d** presents aligned 3D-rendered volumes of the unmixed ElaNIR and HbT signals, together with 3D visualization of the extracted skin layer. These volumetric visualizations enable assessment of the 3D distribution of ElaNIR signals and HbT-defined vascular structures, while also allowing selective extraction of the skin layer to examine its three-dimensional morphology (Movie S1, Supporting Information). Overall, these data demonstrate that 3D multispectral PACT enables spectral separation of ElaNIR signals from vascular HbT signals in 3D volumes, enabling co-registered interpretation of probe biodistribution within an HbT-derived anatomical context.

Supplementary material related to this article can be found online at doi:10.1016/j.pacs.2026.100857.

The following is the Supplementary material related to this article Movie S1.

Movie S1

### 3.3 Whole-body ElaNIR biodistribution

Using the unmixed volumetric images spanning depths of ∼10 mm, we next analyzed the depth-resolved distribution of ElaNIR signals relative to organ-level anatomy. Frontal, sagittal, and transverse cross-sectional images were extracted from pre- and post-injection whole-body datasets. Frontal planes were extracted at an approximate depth of 3 mm from the skin surface, while sagittal and transverse sections were generated at locations corresponding to major organs (indicated by red and yellow dashed lines).

**Fig. 3:**
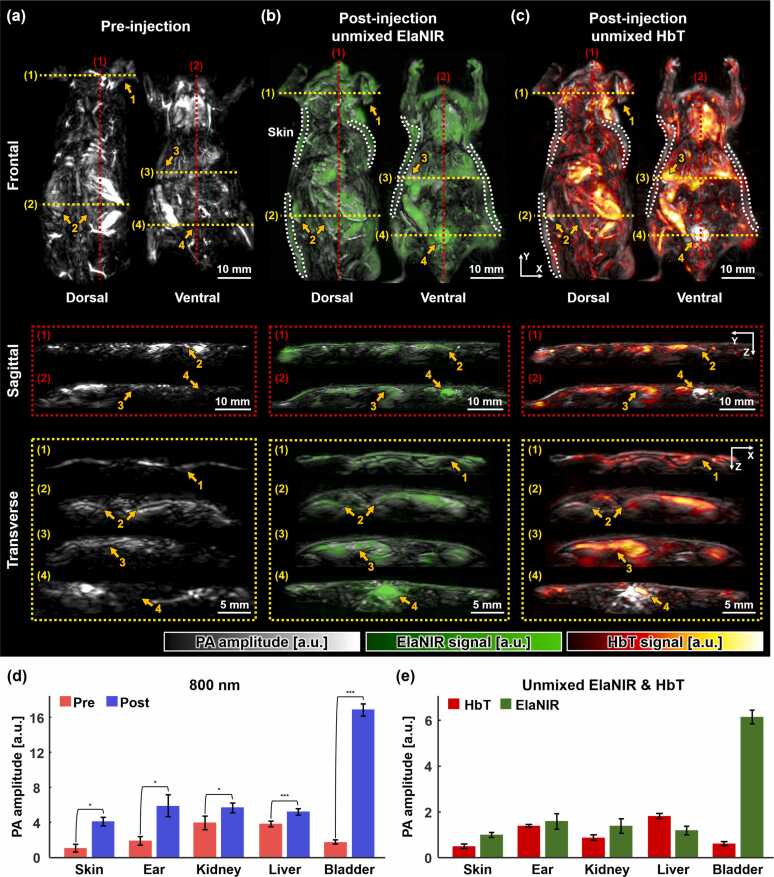
**Cross-sectional analysis of ElaNIR biodistribution in major organs.** (a) Pre-injection cross-sectional images in the frontal, sagittal, and transverse planes shown in both dorsal and ventral views. (b, c) Post-injection spectrally unmixed cross-sectional images of (b) ElaNIR (green) and (c) HbT (hot) at the same locations. Dashed lines in the frontal images indicate the locations of the corresponding sagittal (red dashed box) and transverse (yellow dashed box) sections. White dashed boxes indicate the skin layer. Orange arrows indicate major organs. (d) Quantification of PA signals at 800 nm in the pre-injection and 0 h post-injection (n = 3). Signal intensities were normalized to the pre-injection skin signal. (e) Quantification of unmixed ElaNIR and HbT signals at post-injection (n = 3). Signal intensities were normalized to the ElaNIR signal in the skin. *p < 0.05; **p < 0.01; ***p < 0.005. Error bars represent standard errors. 1, ear; 2, kidney; 3, liver; 4, bladder.

Before ElaNIR injection, cross-sectional images show only vascular and organ structures with negligible PA signals in the skin layers (Fig. 3**a**). After ElaNIR injection, a distinct and continuous signal layer appears along the skin surface (white dashed boxes), with localized signal increases in the ear, kidneys, liver, and bladder in the frontal sections **(**Fig. 3**b)**. Sagittal and transverse sections further corroborate the depth-resolved distribution of ElaNIR signals along the skin and within internal organs. In the corresponding HbT map (Fig. 3**c**), vascular structures and internal organ morphology are clearly delineated. The HbT map shows a minimal signal in the skin layer and bladder, whereas regions with intrinsically high hemoglobin content, such as the ear, kidneys, and liver, remain prominent. This complementary spatial pattern across orthogonal views between ElaNIR and the endogenous hemoglobin background provides anatomical context for interpreting probe distribution and distinguishing ElaNIR-associated signal enhancement from hemoglobin-derived signals. A depth-resolved frontal slice movie confirms the volumetric signal enhancement and biodistribution of the unmixed signals across the whole body (Movie S2, Supporting information).

Supplementary material related to this article can be found online at doi:10.1016/j.pacs.2026.100857.

The following is the Supplementary material related to this article Movie S2.

Movie S2

The observed organ-level patterns are consistent with the reported properties of ElaNIR, including preferential affinity for elastin and renal–urinary clearance after systemic administration. Skin and ear signals likely reflect elastin-associated probe retention. In contrast, signals in the kidneys, liver, and bladder likely encompass a combination of clearance-related dynamics and local elastin-specific retention. In particular, kidney signals may be influenced by both renal clearance and high vascularity [56], whereas bladder signals may arise from urinary probe accumulation as well as the intrinsic elasticity of the tissue [57], [58].

These findings on the 3D whole-body distribution of ElaNIR were also quantitatively analyzed. Fig. 3**d** compares PA signals at 800 nm between pre- and post-injection images, revealing significant increases across the skin and target organs (skin: 3.90 ± 0.47 a.u., p = 0.043; ear: 5.55 ± 1.18 a.u., p = 0.039; kidney: 5.42 ± 0.56 a.u., p = 0.019; liver: 4.98 ± 0.35 a.u., p = 0.0013; bladder: 15.83 ± 0.69 a.u., p = 0.0018). Since ElaNIR exhibits strong absorption near 800 nm, PA amplitude changes at this wavelength provide a sensitive indicator of ElaNIR-associated signal enhancement. This organ-dependent biodistribution is consistently supported by quantitative analysis of the spectrally unmixed ElaNIR and HbT signals at post-injection (Fig. 3**e**). The quantified ElaNIR signals indicate probe presence across target organs and reveal distinct signal levels among them (skin: 1.00 ± 0.10 a.u.; ear: 1.61 ± 0.35 a.u.; kidney: 1.39 ± 0.32 a.u.; liver: 1.21 ± 0.19 a.u.; bladder: 6.15 ± 0.30 a.u.). In contrast, HbT quantification reflects hemoglobin-dominated contrast in the ear, kidneys, and liver while remaining comparatively low in the skin and bladder (skin: 0.50 ± 0.01 a.u.; ear: 1.40 ± 0.06 a.u.; kidney: 0.88 ± 0.12 a.u.; liver: 1.82 ± 0.10 a.u.; bladder: 0.62 ± 0.09 a.u.). Together, the simultaneous quantification of unmixed ElaNIR and HbT components enables assessment of their respective contributions to the total signal enhancement observed at 800 nm. The skin and bladder showed predominantly ElaNIR-driven signal increases, whereas the other organs reflected combined contributions from both ElaNIR accumulation and intrinsic hemoglobin signals. Collectively, these results demonstrate that 3D multispectral PACT serves as a robust tool for both visualizing and quantifying the depth-resolved biodistribution of ElaNIR.

### 3.4 Comparative 3D multispectral PACT in young and old mice

**Fig. 4:**
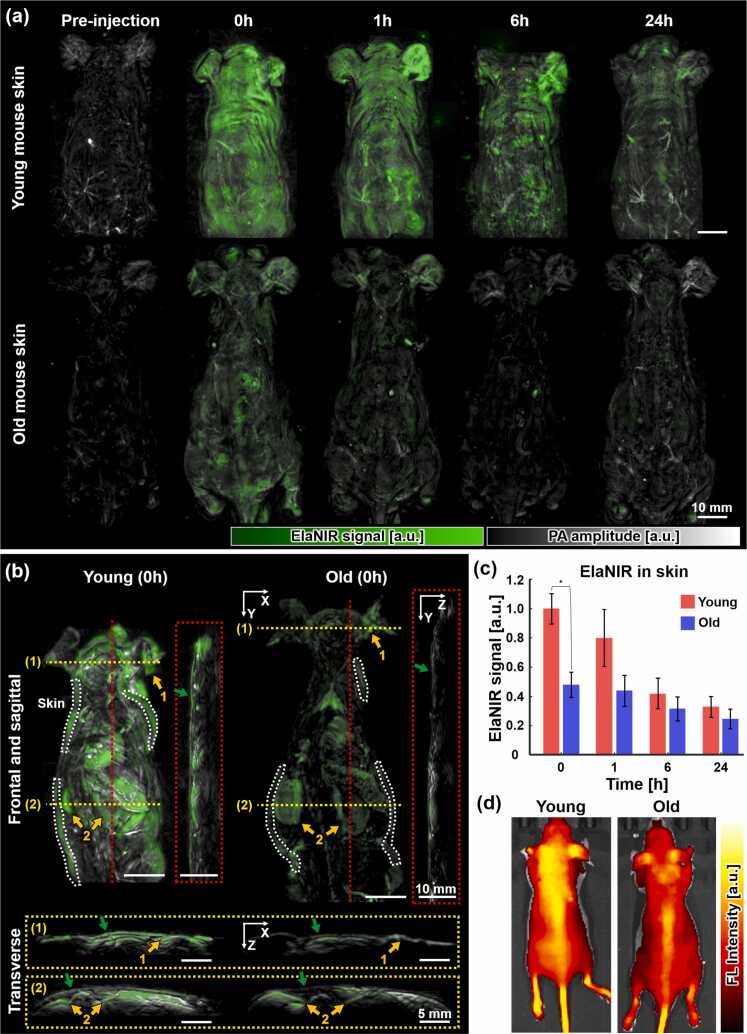
**Whole-Body PACT monitoring of ElaNIR signals in young and old mice.** (a) Skin MAP images of young and old mice in the dorsal plane at pre-injection and 0, 1, 6, and 24 h after ElaNIR injection. (b) Cross-sectional local MAP images in the frontal, sagittal, and transverse planes at 0 h post-injection in young and old mice. The dashed lines in the frontal images indicate the locations of the corresponding sagittal (red dashed box) and transverse (yellow dashed box) sections. White dashed boxes and green arrows indicate skin layer. Orange arrows indicate major organs. (c) Quantification of skin signals from the ElaNIR unmixed volumes in young and old mice (n = 3). Signal intensities were normalized to the 0 h ElaNIR signal of young mice. (d) In vivo FL images acquired using the IVIS system 1 h after injection of ElaNIR. *p < 0.05; **p < 0.01; ***p < 0.005. Error bars represent standard errors. 1, ear; 2, kidney.

Finally, we compared whole-body ElaNIR biodistribution in young (6-week-old) and old (12-month-old) mice over 24 h to investigate age-related physiological alterations. Given that aging is characterized by progressive elastin loss and reduced tissue elasticity [59], [60], we performed longitudinal monitoring pre-injection and at 0, 1, 6, and 24 h post-injection (1 mM ElaNIR, 10 µL/g, retro-orbital). Fig. 4**a** shows longitudinal changes in ElaNIR signals extracted from the segmented dorsal skin layers. Immediately after injection (0 h), young mice exhibit a strong and spatially uniform skin layer, whereas old mice display lower signal intensity with a fragmented distribution **(**Movie S3, Supporting information**)**. These differences persist at 1 h and 6 h and diminish by 24 h as ElaNIR clears and skin signals decrease to similar levels in both groups. The ventral plane shows the same trend, with consistently lower skin signals in old mice (Fig. S4a, Supporting information). Fig. 4**b** shows dorsal whole-body cross-sectional images at 0 h post-injection in three orthogonal planes. Young mice show a distinct, continuous band-like skin signal, in contrast to the discontinuous signals in old mice (white dashed boxes and green arrows). Additionally, images reveal ElaNIR accumulation in the ear and kidneys (orange arrows). These organ-associated signals are also visible in 800 nm whole-body images (Fig. S4b and S5, Supporting information), showing higher post-injection PA signals in the ear, kidneys, liver, and bladder compared with pre-injection images in both groups. Movie S4 **(**Supporting information**)** compares depth-resolved and cross-sectional ElaNIR signals in young and old mice at 0 h and 24 h post-injection, corroborating the time-dependent changes observed in the volumetric and cross-sectional images.

Supplementary material related to this article can be found online at doi:10.1016/j.pacs.2026.100857.

The following is the Supplementary material related to this article Movie S3,Movie S4.

Movie S3

Movie S4

Quantification of skin signal intensity (Fig. 4**c**) confirms significantly higher ElaNIR skin signal in young mice compared with old mice at 0 h (1.00 ± 0.10 a.u. in young and 0.48 ± 0.09 a.u. in the old; *p* = 0.019). This difference persists at 1 h (0.80 ± 0.19 a.u. in young and 0.44 ± 0.11 a.u. in the old). By 6 h post-injection, signals decrease markedly in both groups (0.42 ± 0.10 a.u. in young and 0.32 ± 0.08 a.u. in the old) and continue to decline through 24 h post-injection (0.33 ± 0.07 a.u. in young and 0.25 ± 0.07 a.u. in the old). These results indicate reduced ElaNIR retention in aged skin, consistent with age-related alterations in tissue structure. To validate the multispectral PACT results, we compared ElaNIR signals in both groups using in vivo FL imaging at 1 h post-injection (Fig. 4**d**). Old mice exhibit lower signals in the skin than young mice, consistent with the age-dependent differences observed in PACT.

While conventional FL imaging primarily captures superficial signals, multispectral PACT offers the distinct advantage of depth-resolved 3D information. Leveraging this capability, we quantified ElaNIR signals in selected anatomical regions beyond the skin to assess age-dependent changes in organ-level probe biodistribution (Fig. S6, Supporting information). The ear, an elastin-rich tissue, shows higher ElaNIR signals in young mice than in old mice and follows a temporal trend similar to the skin (young: 1.61 ± 0.35 a.u.–0.38 ± 0.06 a.u.; old: 0.94 ± 0.25 a.u.–0.36 ± 0.08 a.u.). In contrast, signals in the kidneys and liver increase gradually over 24 h (kidneys: young, 1.39 ± 0.32–2.36 ± 0.21 a.u.; old, 1.65 ± 0.47–3.05 ± 0.31 a.u.; liver: young, 1.21 ± 0.19–1.55 ± 0.11 a.u.; old, 1.29 ± 0.13–1.93 ± 0.20 a.u.). In the bladder, ElaNIR signals diminish by 6 h post-injection (0 h: 6.15 ± 0.30 a.u. in the young and 8.48 ± 0.78 a.u. in the old; 6 h: 0.94 ± 0.60 a.u. and 0.64 ± 0.24 a.u.). Notably, signals in the kidneys, liver, and bladder are higher in old mice after injection. The opposite trends observed in elastin-rich tissues and clearance-related organs provide important context for interpreting the age-dependent ElaNIR signal patterns. The reduced skin and ear signals in old mice suggest decreased elastin-associated probe retention, whereas the higher kidney, liver, and bladder signals indicate increased organ-level redistribution and clearance-related accumulation. In other words, a larger fraction of ElaNIR likely remains unbound in old mice, leading to increased systemic circulation and clearance of the free probe. In addition, age-related changes in renal and hepatic clearance function may also contribute to the elevated signals observed in these clearance-related organs [61], [62]. This interpretation is also supported by the reported pharmacokinetic behavior of ElaNIR, in which more than 90% of the injected dose is eliminated from plasma within 4 h after systemic administration [44]. Collectively, these results demonstrate that 3D multispectral PACT enables whole-body longitudinal assessment of ElaNIR signals, integrating direct visualization of probe retention in elastin-rich tissues with pharmacokinetic biodistribution in clearance-related organs to provide depth-resolved context for interpreting age‑related changes in elastin‑associated ElaNIR signal patterns.

## 4 Discussion and conclusion

Elastin is a key ECM component that maintains tissue elasticity and structural integrity, and its remodeling is closely associated with functional alterations observed during aging and in various diseases. In particular, given that elastin regeneration is negligible in adulthood, in vivo alterations in elastin distribution can serve as critical indicators reflecting cumulative tissue remodeling and functional decline. Accordingly, imaging techniques that enable spatially resolved and quantitative assessment of elastin-associated signals are essential for investigating aging- and disease-associated tissue remodeling.

In this study, we combine an NIR elastin-specific probe, ElaNIR, with 3D multispectral PACT to enable depth-resolved volumetric assessment of ElaNIR signals in vivo. Phantom imaging and sensitivity tests validate that ElaNIR provides stable, wavelength-dependent PA contrast with high detection sensitivity and a concentration-dependent signal response, supporting its suitability for PACT-based elastin imaging. In vivo, we administered ElaNIR and performed multispectral PACT to spectrally unmix ElaNIR and HbT signals, allowing ElaNIR distribution to be interpreted with HbT-based anatomical context. Furthermore, cross-sectional and volumetric analyses revealed a continuous ElaNIR signal band along the skin layer and distinct signal enhancement in the ear, kidneys, liver, and bladder. The observed signal patterns can be more appropriately interpreted by considering both the elastin affinity and pharmacokinetic behavior of ElaNIR. In elastin-rich tissues such as the skin and ear, ElaNIR signals likely reflect elastin-associated probe retention, supported by the absence of comparable signals in CyZW−274 control imaging. In contrast, signals in the kidneys, liver, and bladder are unlikely to arise solely from elastin-bound probe. These organs are strongly influenced by systemic probe biodistribution and clearance-related processes. Kidney signals may reflect both renal clearance and high vascularity, whereas bladder signals may primarily include urinary probe accumulation, with possible contribution from the elastic nature of bladder tissue. Therefore, the signals observed in these organs are mixed readouts that include probe pharmacokinetics, vascular or tissue structure, and potential elastin-associated retention. This integrated interpretation is particularly useful for monitoring age-dependent alterations. Young mice exhibit strong and spatially uniform ElaNIR signals in the skin and ear, whereas aged mice show reduced and discontinuous signals. These findings are consistent with age-related alterations in elastin-rich tissue architecture, including reduced elastin availability or altered elastic fiber organization. Conversely, aged mice showed higher ElaNIR signals in clearance-related organs, including the kidneys, liver, and bladder. The opposite trends between elastin-rich tissues and clearance-related organs suggest that reduced tissue retention in aged mice may increase the fraction of unbound or weakly retained ElaNIR, leading to enhanced systemic redistribution and clearance. Accordingly, organ-level biodistribution provides complementary pharmacokinetic context for interpreting age-dependent ElaNIR retention patterns in elastin-rich tissues. Compared with conventional FL imaging, 3D multispectral PACT provides depth-resolved volumetric information and co-registered anatomical context. FL imaging confirmed lower skin ElaNIR signals in aged mice, consistent with the PACT results, but it primarily captured superficial signal differences. In contrast, multispectral PACT enabled simultaneous visualization of skin and ear signals, internal organ biodistribution, and HbT-defined vascular anatomy within a single volumetric dataset. Thus, age-dependent ElaNIR signal changes could be interpreted not only as superficial tissue differences, but also in relation to systemic probe redistribution and clearance. Overall, 3D multispectral PACT provides a more integrated in vivo framework for elastin-related studies than surface-weighted optical imaging alone.

However, several aspects of this study could be further improved in future research. First, although previous studies and our CyZW−274 control results support the preferential affinity of ElaNIR for elastin-rich tissues, the present study does not directly distinguish elastin-bound ElaNIR from unbound or circulating ElaNIR. In particular, the kidney, liver, and bladder signals may include substantial pharmacokinetic and clearance-related contributions. Therefore, we interpret the measured PA signals as ElaNIR-associated signals rather than direct quantitative maps of elastin content. Nonetheless, the observed biodistribution and clearance patterns offer a highly informative functional baseline for tracking the agent's behavior within a complex living system. Additional validation, such as organ-specific histological comparison after vascular perfusion [63], elastase-treated tissue controls [64], competition assays [65] would further strengthen the molecular interpretation of ElaNIR retention in specific tissues. Second, the accuracy of spectral unmixing can be challenged under complex in vivo conditions. In this study, the ElaNIR spectrum was estimated from wavelength-dependent signals in the bladder region, which was selected for its relatively low hemoglobin contribution and probe-dominant contrast. Nevertheless, because optical attenuation in biological tissue is wavelength- and depth-dependent, the effective local fluence spectrum may vary across anatomical sites. As a result, applying a fixed in vivo spectrum globally may introduce uncertainty in the apparent spectral profile and signal amplitude used for spectral unmixing, particularly when comparing ElaNIR signals between superficial tissues and deeper internal organs. Therefore, signal differences between superficial and deep tissues should be interpreted with consideration of potential fluence-related effects. However, the main analyses in this study focused on relative group differences and longitudinal trends acquired under consistent imaging and analysis conditions, which helps reduce the impact of systematic biases on comparative conclusions. Future work could mitigate these limitations by implementing fluence-compensation strategies [66], [67], [68], performing mixed-absorber phantom validation [69], [70], or utilizing advanced unmixing algorithms such as blind source separation [55], [71], [72]. Furthermore, the use of three excitation wavelengths may be affected by spectral overlap between ElaNIR and endogenous chromophores. Incorporating additional excitation wavelengths in future studies could provide more spectral information and further improve the robustness and accuracy of spectral unmixing [71], [73], [74]. In addition, although the sample size was determined based on an a priori power analysis, future studies with larger datasets would further strengthen the reproducibility and generalizability of these findings. Finally, imaging depth is inherently constrained by the fundamental limitations of optical excitation in PACT [75], [76]. As depth increases, optical attenuation and scattering become more pronounced, potentially reducing the signal-to-noise ratio and compromising quantitative accuracy in deep tissues [77]. Although the imaging depth achieved in this study was sufficient for assessing skin elastin and adjacent major organs, further improvements in depth performance and signal stability may be achieved by using higher-power laser source [78], optimizing light delivery [79], and implementing adaptive fluence-compensation techniques [80], [81].

## Author Contributions

H.J., J.K., H-Y.K., C.K., Y-T.J., and N-Y.K. contributed to the conceptualization of the study. H.J. and J.K. performed the overall experiments and data analysis. H-Y.K. and N-Y.K. prepared the probe, performed probe characterization, and established the animal models. J.L. and W.K. conducted IVIS FL imaging to validate the PACT results. The project was supervised by C.K., Y-T.J., and N-Y.K. All authors were involved in discussing the results and writing the manuscript.

## CRediT authorship contribution statement

**Jihye Lee:** Validation, Resources. **Won Jong Kim:** Validation, Resources. **Nam-Young Kang:** Supervision, Project administration, Conceptualization. **Young-Tae Chang:** Writing – review & editing, Supervision, Project administration, Conceptualization. **Chulhong Kim:** Writing – review & editing, Supervision, Project administration, Conceptualization. **Hyunseo Jeon:** Writing – review & editing, Writing – original draft, Software, Project administration, Methodology, Investigation, Data curation, Conceptualization. **Jiwoong Kim:** Writing – review & editing, Writing – original draft, Software, Project administration, Methodology, Investigation, Data curation, Conceptualization. **Haw-Young Kwon:** Writing – review & editing, Writing – original draft, Project administration, Methodology, Investigation, Conceptualization.

## Declaration of Competing Interest

The authors declare the following financial interests/personal relationships which may be considered as potential competing interests. Chulhong Kim reports financial support was provided by National Research Foundation of Korea. Chulhong Kim reports financial support was provided by Korea Health Industry Development Institute. Chulhong Kim reports financial support was provided by Commercializations Promotion Agency for R&D Outcomes. Chulhong Kim reports financial support was provided by Institute of Information & Communications Technology Planning & Evaluation. Chulhong Kim reports financial support was provided by BK21 Four program. Chulhong Kim reports financial support was provided by Glocal University 30 project. Young-Tae Chang reports financial support was provided by Basic Science Research Institute Fund. Young-Tae Chang reports financial support was provided by Starting Growth Technological R&D Program (TIPS Program). Hyunseo Jeon reports financial support was provided by Hyundai Motor Chung Mong-Koo Foundation. Young-Tae Chang reports financial support was provided by National Research Foundation of Korea. Nam-Young Kang reports financial support was provided by National Research Foundation of Korea. Haw-Young Kwon reports financial support was provided by National Research Foundation of Korea. If there are other authors, they declare that they have no known competing financial interests or personal relationships that could have appeared to influence the work reported in this paper.

## Data Availability

Data will be made available on request.
