# Supplementary material for: Volumetric photoacoustic imaging of elastin and age-related remodeling using near-infrared probe ElaNIR
Source: Photoacoustics. 2026 Jul 18;51:100857. doi: 10.1016/j.pacs.2026.100857 (PMC13393824; doi:10.1016/j.pacs.2026.100857)
Supplement: Supplementary file 5 — Supplementary material [file mmc1.docx]

**Volumetric Photoacoustic Imaging of Elastin and Age-related Remodeling Using Near-infrared Probe ElaNIR**

*Hyunseo Jeon^1†^, Jiwoong Kim^1†^, Haw-Young Kwon^2,4†^, Jihye Lee^2^, Won Jong Kim^2^, Nam-Young Kang^3,4^*, Young-Tae Chang^2^*, and Chulhong Kim^1^**

^1^Department of Electrical Engineering, Convergence IT Engineering, Medical Science and Engineering, Institute of Artificial Intelligence, and Medical Device Innovation Center, Pohang University of Science and Technology, Republic of Korea

^2^Department of Chemistry, Pohang University of Science and Technology (POSTECH), Pohang 37673, Republic of Korea

^3^Department of Convergence IT Engineering, Pohang University of Science and Technology (POSTECH), Pohang 37673, Republic of Korea

^4^SenPro, C5 building, Pohang University of Science and Technology, Pohang, Gyeongbuk 37673, Korea

†These authors contributed equally to this work.

*E-mails: [chulhong@postech.edu](mailto:chulhong@postech.edu), [ytchang@postech.ac.kr](mailto:ytchang@postech.ac.kr), [knysg@postech.ac.kr](mailto:knysg@postech.ac.kr)

| **Supplementary Figure 1** | Schematic of 3D multispectral PACT imaging system. |
| --- | --- |
| **Supplementary Figure 2** | Spectral unmixing based on the estimated ElaNIR PA spectrum. |
| **Supplementary Figure 3** | Control PACT imaging of CyZW-274. |
| **Supplementary Figure 4** | Ventral plane whole-body PACT monitoring in young and old mice. |
| **Supplementary Figure 5** | Dorsal plane whole-body PACT monitoring in young and old mice. |
| **Supplementary Figure 6** | Changes in ElaNIR signals over 24 h in major organs of young and old mice. |
| **Supplementary Movie 1** | 3D volumetric analysis of unmixed ElaNIR and HbT signals. |
| **Supplementary Movie 2** | Frontal cross-sectional analysis of ElaNIR injected mouse |
| **Supplementary Movie 3** | Skin signal comparison between young and old mice. |
| **Supplementary Movie 4** | Cross-sectional ElaNIR signal comparison between young and old mice. |


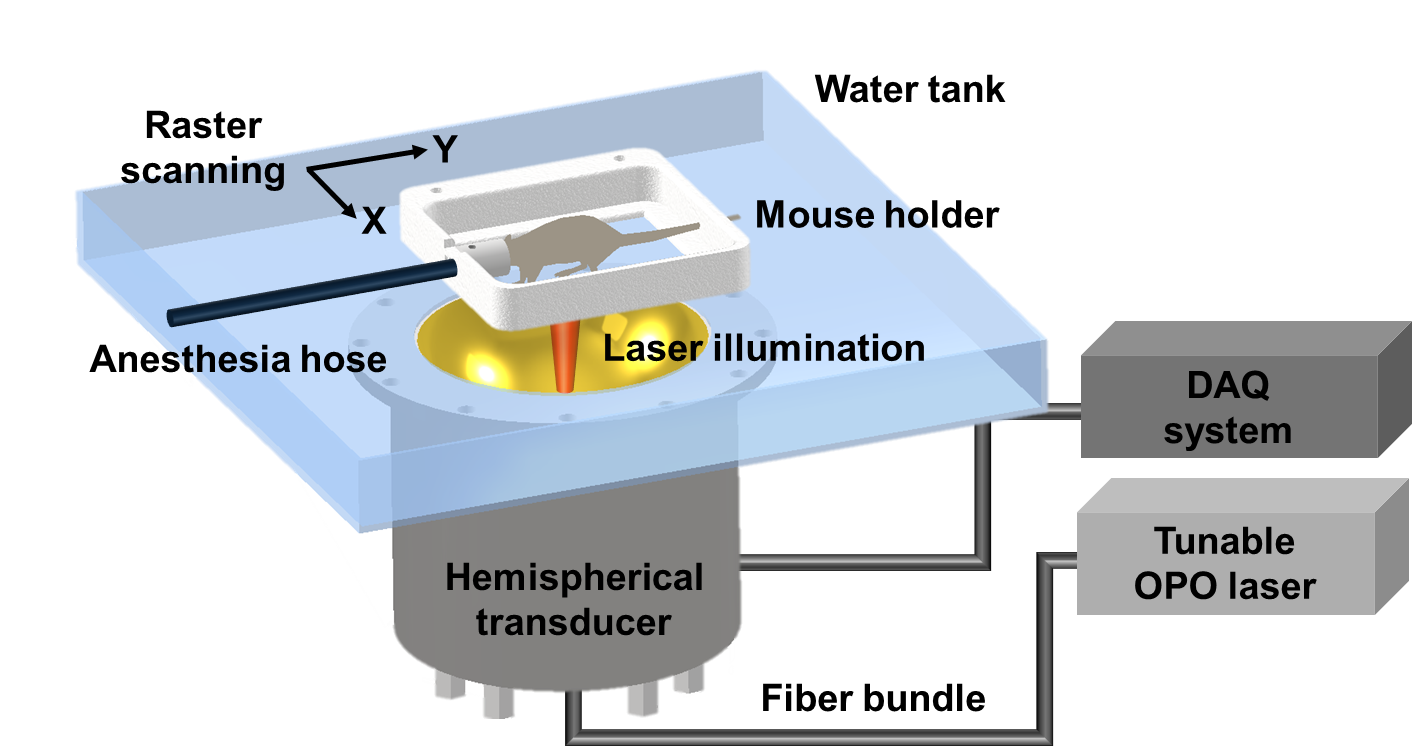


**Supplementary Figure 1. Schematic of 3D multispectral PACT imaging system.** The system consists of a 1024-element hemispherical ultrasound transducer array and a tunable OPO laser for multispectral excitation. The mouse is placed on a custom holder and raster-scanned in the XY plane using a motorized stage. DAQ, data acquisition; OPO, optical parametric oscillator.


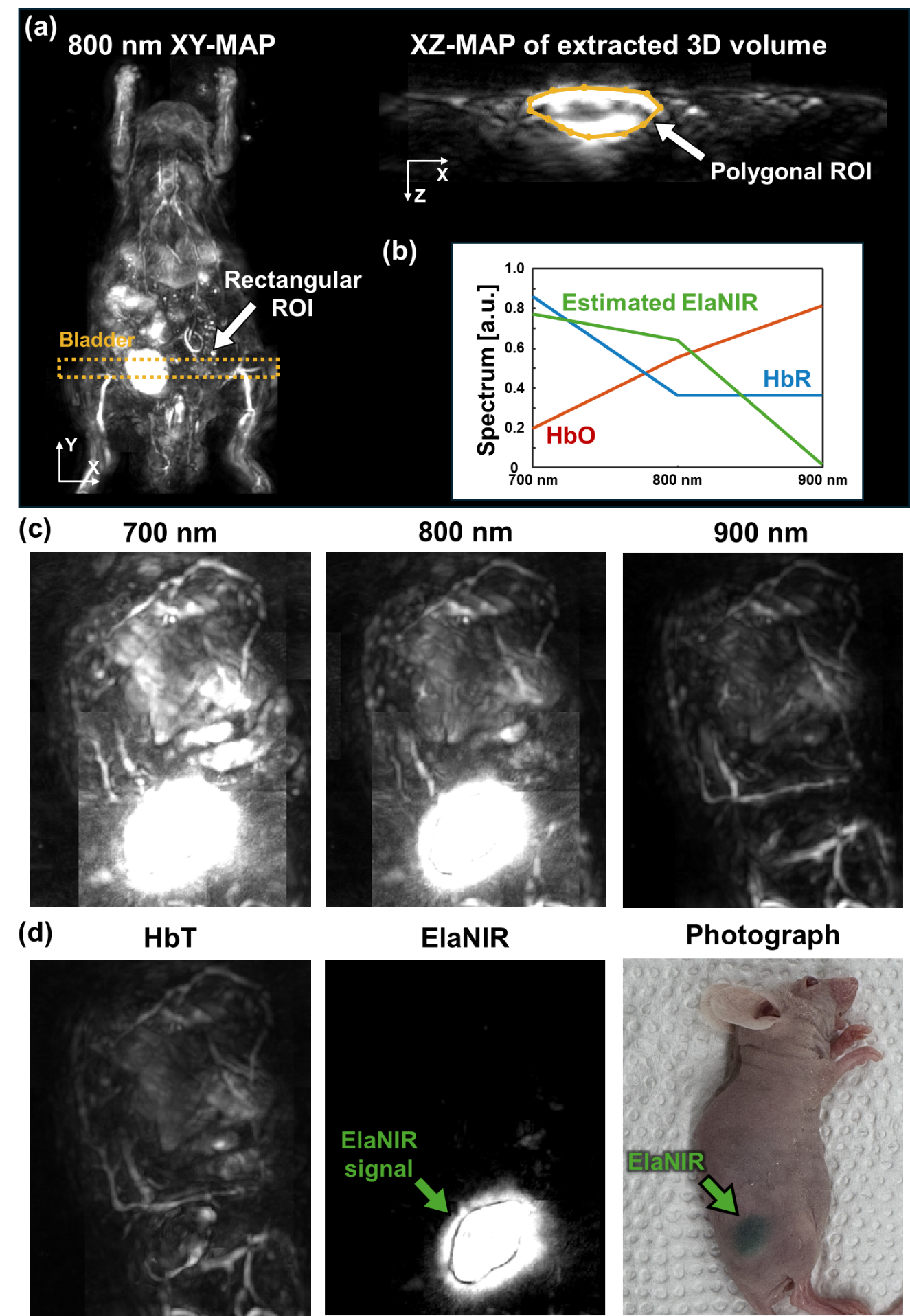


**Supplementary Figure 2. Spectral unmixing based on the estimated ElaNIR PA spectrum.** (a) Definition of 3D ROI for the bladder. (b) Unmixing spectra derived from ROI-based signal quantification. (c) Multispectral PACT images acquired in the right sagittal plane at 700, 800, and 900 nm. (d) Spectral unmixing results of subcutaneously injected ElaNIR.

**
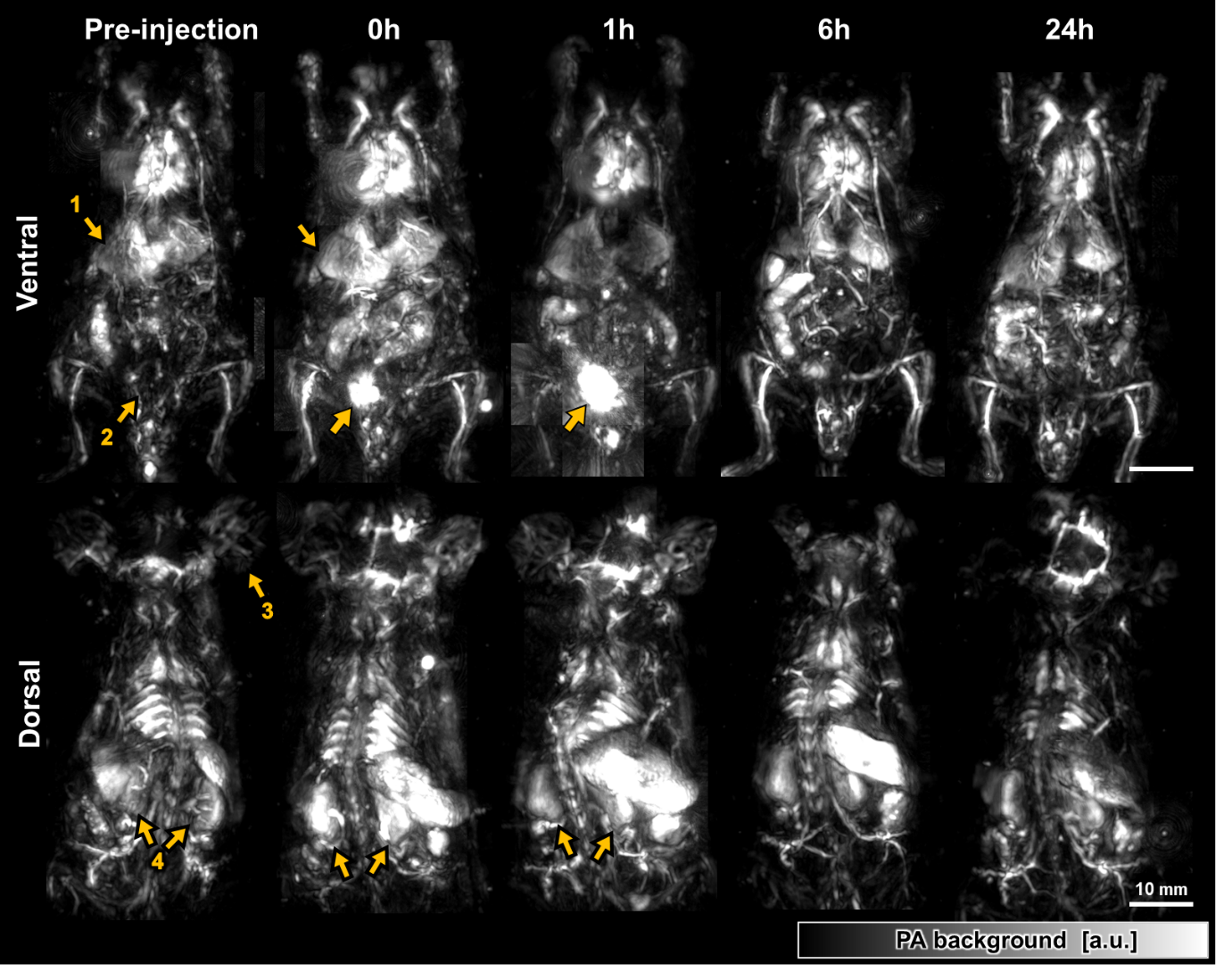
**

**Supplementary Figure 3.** **Control PACT imaging of CyZW-274.** MAP images at 800 nm in the dorsal and ventral planes acquired before injection and at 0, 1, 6, and 24 h after injection of CyZW-274, a structurally analogous control dye with low elastin affinity. Orange arrows indicate major organs. 1, liver; 2, bladder; 3, ear; 4, kidney.


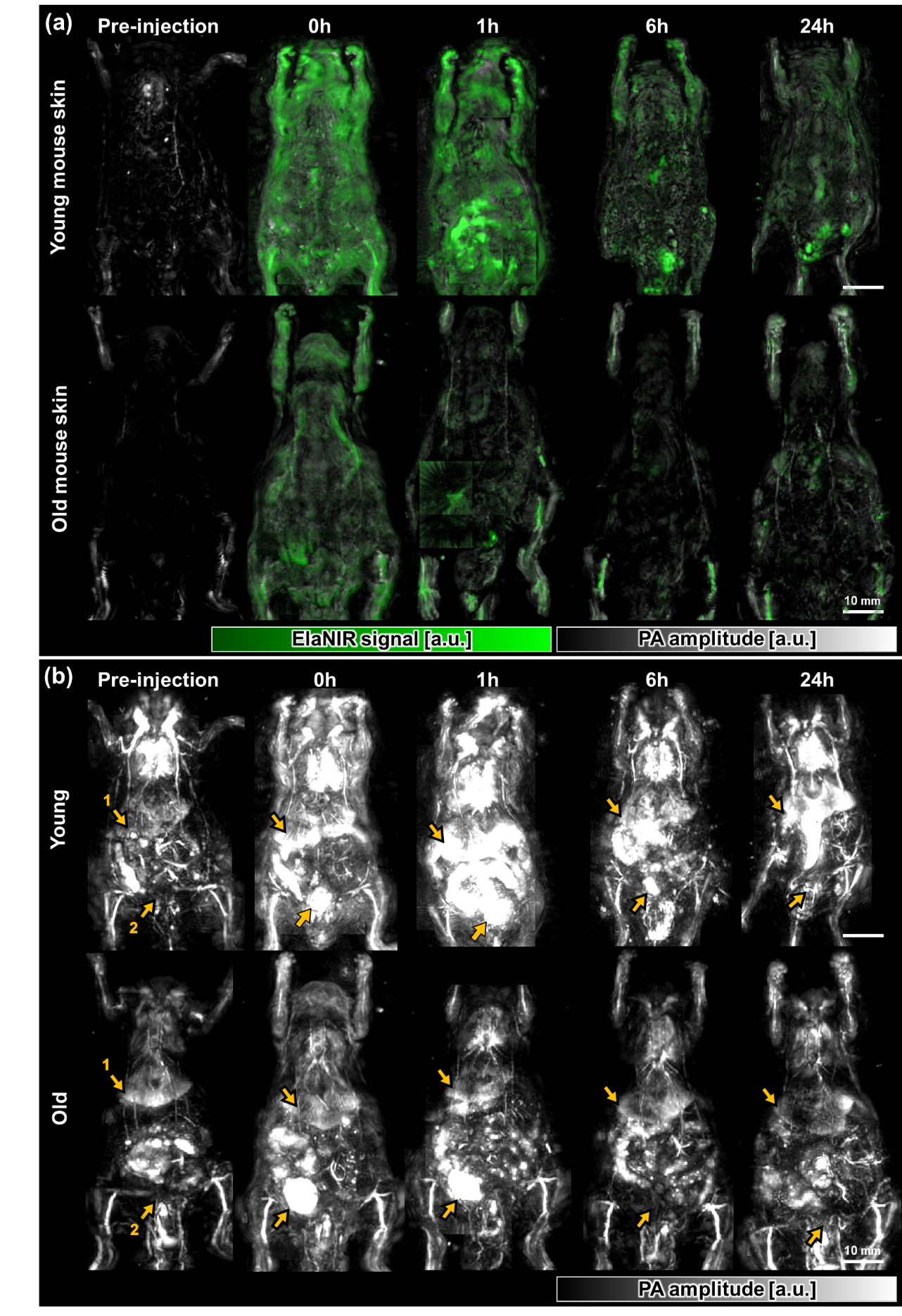


**Supplementary Figure 4.** **Ventral plane whole-body PACT monitoring in young and old mice.** (a) Skin MAP images of young and old mice in the ventral plane at pre-injection and 0, 1, 6, and 24 h after ElaNIR injection. (b) MAP images at 800 nm of young and old mice in the ventral plane at pre-injection and 0, 1, 6, and 24 h after ElaNIR injection. Orange arrows indicate major organs. 1, liver; 2, bladder.


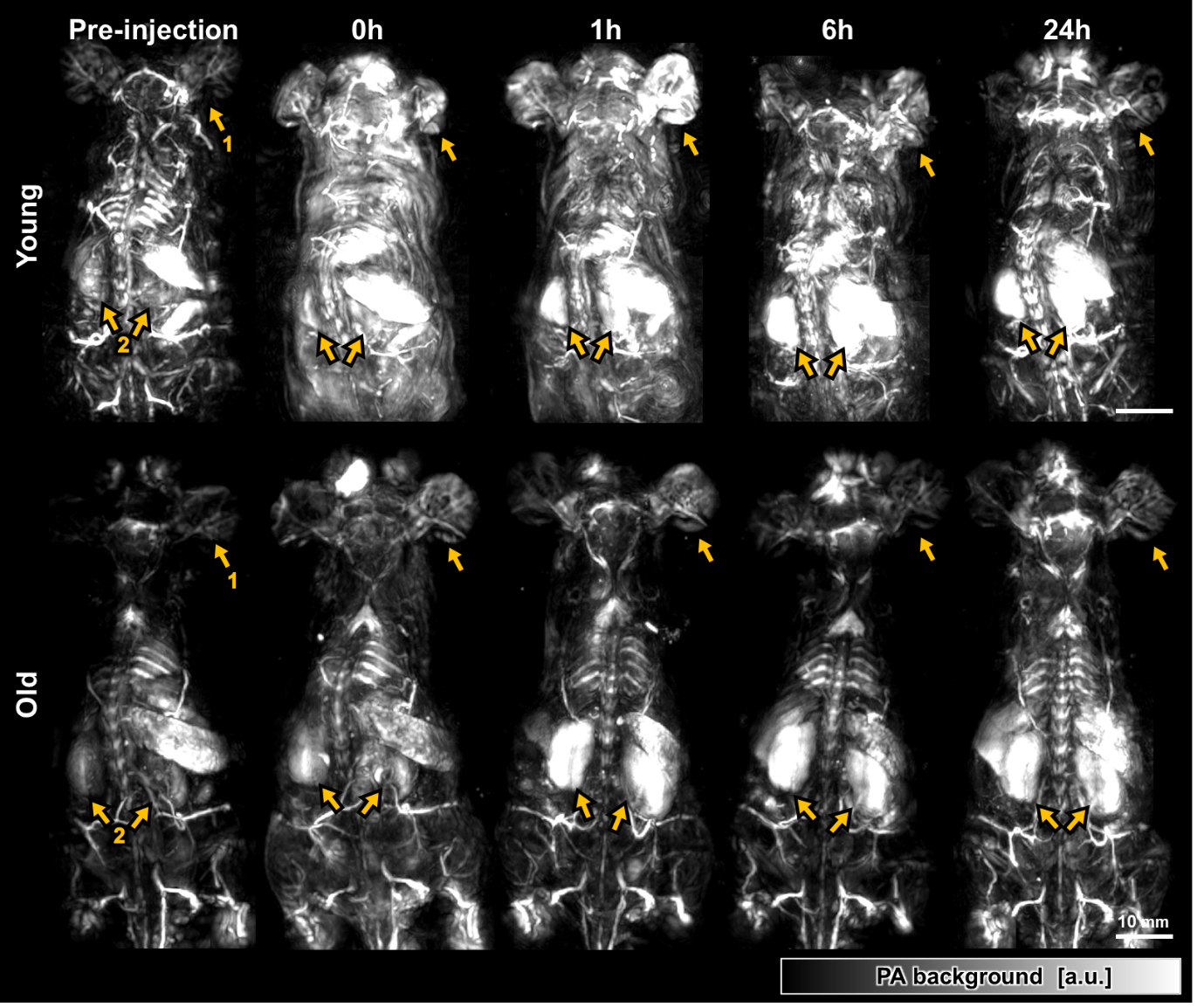


**Supplementary Figure 5.** **Dorsal plane whole-body PACT monitoring in young and old mice.** MAP images at 800 nm of young and old mice in the dorsal plane at pre-injection and 0, 1, 6, and 24 h after ElaNIR injection. Orange arrows indicate major organs. 1, ear; 2, kidney.


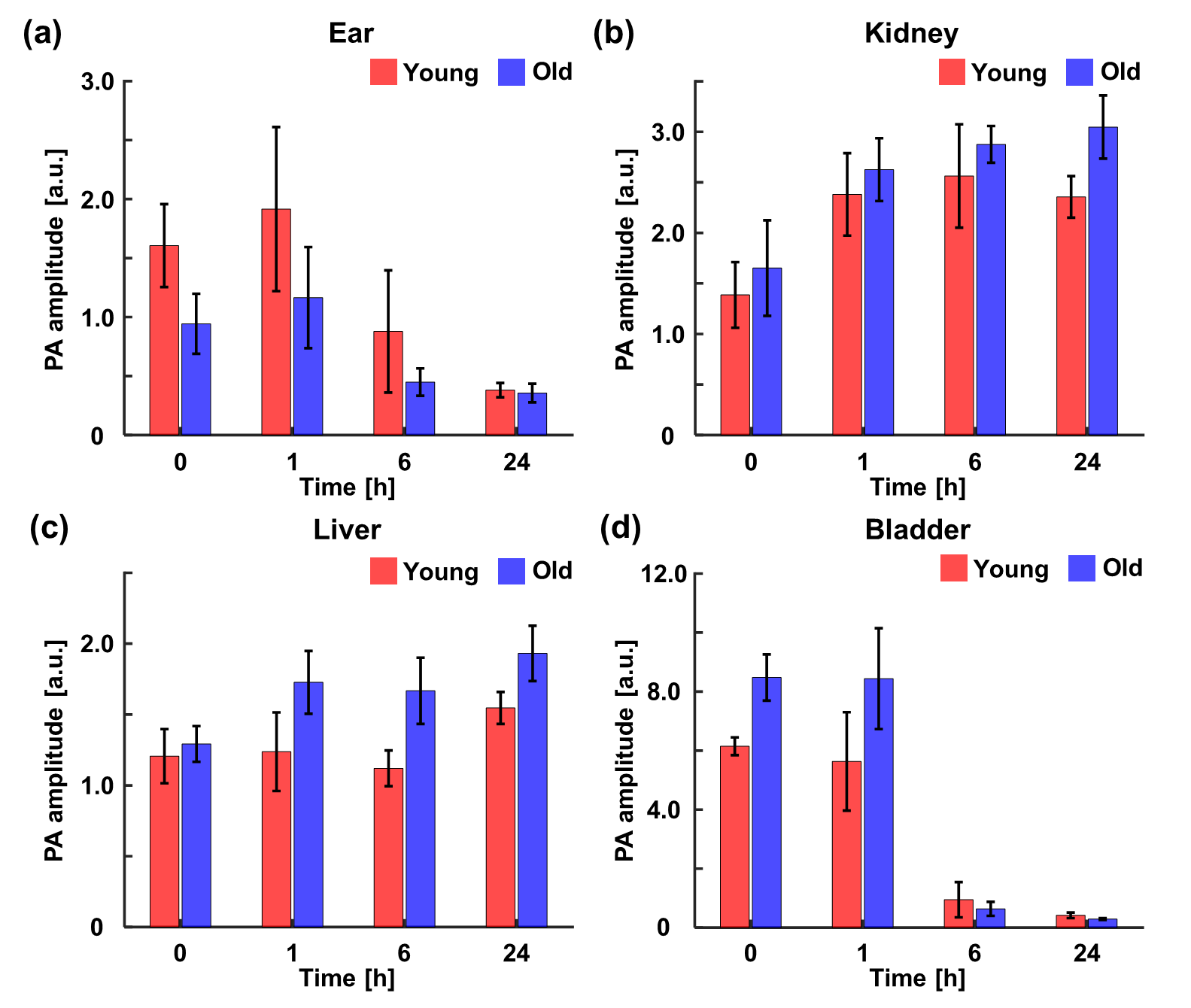


**Supplementary Figure 6.** **Changes in ElaNIR signals over 24 h in major organs of young and old mice**. (a) Ear, (b) kidney, (c) liver, and (d) bladder. Signal intensities were normalized to the 0 h ElaNIR signal in the skin of young mice. Error bars represent standard errors (n = 3).
